# Vital Signs Prediction and Early Warning Score Calculation Based on Continuous Monitoring of Hospitalised Patients Using Wearable Technology

**DOI:** 10.3390/s20226593

**Published:** 2020-11-18

**Authors:** Ahmed Youssef Ali Amer, Femke Wouters, Julie Vranken, Dianne de Korte-de Boer, Valérie Smit-Fun, Patrick Duflot, Marie-Hélène Beaupain, Pieter Vandervoort, Stijn Luca, Jean-Marie Aerts, Bart Vanrumste

**Affiliations:** 1 E-MEDIA, STADIUS, Department of Electrical Engineering (ESAT), Campus Group T, KU Leuven, 3000 Leuven, Belgium; Ahmed.youssefaliamer@kuleuven.be; 2 Measure, Model & Manage Bioresponses (M3-BIORES), Department of Biosystems, KU Leuven, 3000 Leuven, Belgium; jean-marie.aerts@kuleuven.be; 3 Limburg Clinical Research Center/Mobile Health Unit, Faculty of Medicine and Life Sciences, Hasselt University, 3500 Hasselt, Belgium; femke.wouters@uhasselt.be (F.W.); julie.vranken@uhasselt.be (J.V.); pieter@vandervoort.mobi (P.V.); 4 Limburg Clinical Research Center/Mobile Health Unit, Department of Anesthesiology, Department of Cardiology and Department Future Health, Ziekenhuis Oost-Limburg, 3600 Genk, Belgium; 5 Department of Anesthesiology and Pain Management, Maastricht UMC+, 6229 HX Maastricht, The Netherlands; dianne.de.korte@mumc.nl (D.d.K.-d.B.); v.smit.fun@mumc.nl (V.S.-F.); 6 Service des Applications Informatiques, Centre Hospitalier Universitaire de Liège—CHU, 4000 Liège, Belgium; pduflot@chuliege.be; 7 Unité de Pneumologie—Cardiologie—radiothéRapie, Centre Hospitalier Universitaire de Liège—CHU, 4000 Liège, Belgium; Marie-Helene.Beaupain@chuliege.be; 8 Department of Data Analysis and Mathematical Modelling, Ghent University, 9000 Ghent, Belgium; stijn.luca@ugent.be

**Keywords:** vital signs, early warning score, time-series prediction, kNN-LS-SVM, wearable technology

## Abstract

In this prospective, interventional, international study, we investigate continuous monitoring of hospitalised patients’ vital signs using wearable technology as a basis for real-time early warning scores (EWS) estimation and vital signs time-series prediction. The collected continuous monitored vital signs are heart rate, blood pressure, respiration rate, and oxygen saturation of a heterogeneous patient population hospitalised in cardiology, postsurgical, and dialysis wards. Two aspects are elaborated in this study. The first is the high-rate (every minute) estimation of the statistical values (e.g., minimum and mean) of the vital signs components of the EWS for one-minute segments in contrast with the conventional routine of 2 to 3 times per day. The second aspect explores the use of a hybrid machine learning algorithm of kNN-LS-SVM for predicting future values of monitored vital signs. It is demonstrated that a real-time implementation of EWS in clinical practice is possible. Furthermore, we showed a promising prediction performance of vital signs compared to the most recent state of the art of a boosted approach of LSTM. The reported mean absolute percentage errors of predicting one-hour averaged heart rate are 4.1, 4.5, and 5% for the upcoming one, two, and three hours respectively for cardiology patients. The obtained results in this study show the potential of using wearable technology to continuously monitor the vital signs of hospitalised patients as the real-time estimation of EWS in addition to a reliable prediction of the future values of these vital signs is presented. Ultimately, both approaches of high-rate EWS computation and vital signs time-series prediction is promising to provide efficient cost-utility, ease of mobility and portability, streaming analytics, and early warning for vital signs deterioration.

## 1. Introduction

Monitoring of vital signs of hospitalised patients is of paramount importance to deliver timely and adequate care. Numerous studies that focus on analysing vital signs hypothesise that many adverse events are preceded with a disruption in the vital signs [1,2,3,4,5,6]. Monitoring is conventionally achieved via expensive and cumbersome devices [7]. In addition, these conventional monitoring devices have limited mobility and portability.In hospitals, early warning score (EWS) systems are used to indicate deterioration of the vital signs heart rate, respiration rate, systolic blood pressure, oxygen saturation, and temperature [8]. One limitation of this EWS is that it evaluates the current instantaneous measurement of the vital sign but provides no past trends or future predictions of vital signs. Another limitation is the low frequency of observations in clinical practice which is typically between two to three times a day [9]. This relatively low frequency results from monitoring vital signs with cumbersome devices in combination with manual recording of the EWS by the nurses (e.g., respiration rate).

Therefore, continuous monitoring of the vital signs of hospitalised patients using wearable technology is expected to overcome the limitation of the conventional low-rate measurement of EWS at the hospital. Furthermore, continuous monitoring of vital signs provides the medical staff a more complete picture and clinical insight into the patients’ health status and progression [10]. Motivated by these challenges, we aim in this paper to develop a model for predicting future values of vital signs in addition to continuously monitoring of EWS using wearable technology. For critical care patients, predicting any adverse events based on vital signs analysis is investigated exhaustively in several studies such as sepsis prediction [11] and mortality prediction [12]. However, for general wards, we hypothesise that predicting the future value of vital signs can provide early detection of any deterioration of the patients’ health state. Moreover, due to the difficulty of obtaining a real-time annotation/labelling of the monitored vital signs, we have to predict values instead of labels or scores. An important aspect in this regard is the recording frequency since it influences the magnitude of the prediction horizon. Recently Shiyu Liu et al. [13] proposed a generative boosting approach of long-short term memory (LSTM) deep neural networks to predict the vital signs values for specific prediction horizons (up to three hours ahead). Their dataset included demographic data and vital signs of 177 medical patients (nonspecific ward) at regular intervals of 5 min over 24 h. Moreover, they used a mutual information-based clustering algorithm to select a more representative dataset to train the generative model. To the authors’ knowledge this study describes one of the most performant algorithms in the state of the art for predicting vital signs. More specifically, they predicted heart rate and systolic blood pressure 20 min in advance, with a mean absolute percentage error of 7.41 and 6.17%, respectively.

In our study, we aim at investigating two aspects of monitoring vital signs and EWS using wearable technology. Firstly, we aim at computing and monitoring the vital signs components of the EWS at a high-rate (i.e., every minute). Secondly, we aim at predicting at least one-hour ahead statistical attributes of the different vital signs (i.e., minimum, maximum, mean). The latter approach is achieved by using the localised learning approach of KNN-LS-SVM. The KNN-LS-SVM approach is chosen to provide an online prediction with more personalised characteristics of the model [14]. To the authors’ knowledge, it is the first time to apply a localised learning algorithm (i.e., kNN-LS-SVM) to predict vital-signs time series. It is worth mentioning that kNN-LS-SVM for regression is proposed before in the studies of [15,16,17] as a continuation of the fundamental study of local learning algorithms [18].

This article is arranged as follows; after the introduction section, an overview of the EAGLE study is introduced in Section 2. Then, Section 3 is about the methods that are going to be applied to analyse the vital signs for the two proposed approaches. In Section 4, the results of the two proposed approaches are illustrated. Next we discuss our findings in Section 5. Finally, the conclusion of this study is presented in Section 6.

## 2. Data Generation

The EWS is a scoring system implemented in many care centres worldwide, which helps to prevent clinical deterioration of the patients at general wards, via early recognition of disease worsening (i.e., change in one or more vital parameters) [8,19,20,21,22]. In detail, essential vital parameters (heart rate, respiration rate, blood pressure, temperature, oxygen saturation, and neurological responsiveness) are recorded multiple times a day. However, the classic EWS system has its limitations as mentioned earlier in the introduction.

The EWS scoring system has already been proven to be an effective tool in reducing clinical deterioration, reducing the admission to intensive care units and thus overall reducing mortality [19,23,24]. However, as mentioned above, the EWS is measured in clinical practice at a rather low frequency. Therefore, estimation of the EWS score via continuously monitored parameters is expected to further increase patient survival.

The data in this work were generated in the framework of the “The new gold standard: the Early Warning Score algorithm” (i.e., EAGLE) study, which was part of the Interreg EMR project WearIT4health on the development of wearables for hospitalised patients. The objectives of the EAGLE study are: (1) to collect continuously monitored vital parameter data using wearable sensors of patients admitted at the general ward. (2) To develop an algorithm that can early identify clinical deterioration and can optimise the application of the conventional EWS system, and (3) to explore the possibility to predict the measures of the vital signs and EWS for specific prediction horizons.

### 2.1. Study Design

The EAGLE study is an international, multicentre, prospective, and interventional study that includes the following study sites: Centre Hospitalier Universitaire de Liège (CHU) at Liège, Belgium. Ziekenhuis Oost-Limburg (ZOL) at Genk, Belgium and Academisch Ziekenhuis Maastricht (aZM) at Maastricht, The Netherlands. The data collection is done according to the study protocol in accordance with GCP and ICH guidelines. The study was approved by the ethical commission of CHU (B707201836800) and aZM.

### 2.2. Study Population

Patients of the participating hospitals were selected taking into account the following exclusion criteria: (i) eligible patients should not be younger than 18 years old, (ii) eligible patients should not be recruited from high-intensity units (i.e., intensive care units, coronary care units and emergency rooms), (iii) eligible patients should not participate in other studies that might influence the study results (e.g., experimental medication that could affect the heart rate), (iv) eligible patients should not be hospitalised for a 1 day clinic stay, (v) eligible patients should not have infectious diseases.

This resulted in the following target populations: 52 cardiology patients at ZOL and 21 at CHU (on average 23 h × 2 days), 10 postsurgical patients at aZM (on average 23 h × 1 day), and 7 dialysis patients at CHU (on average 4 h × 2 sessions).

**Figure 1:**
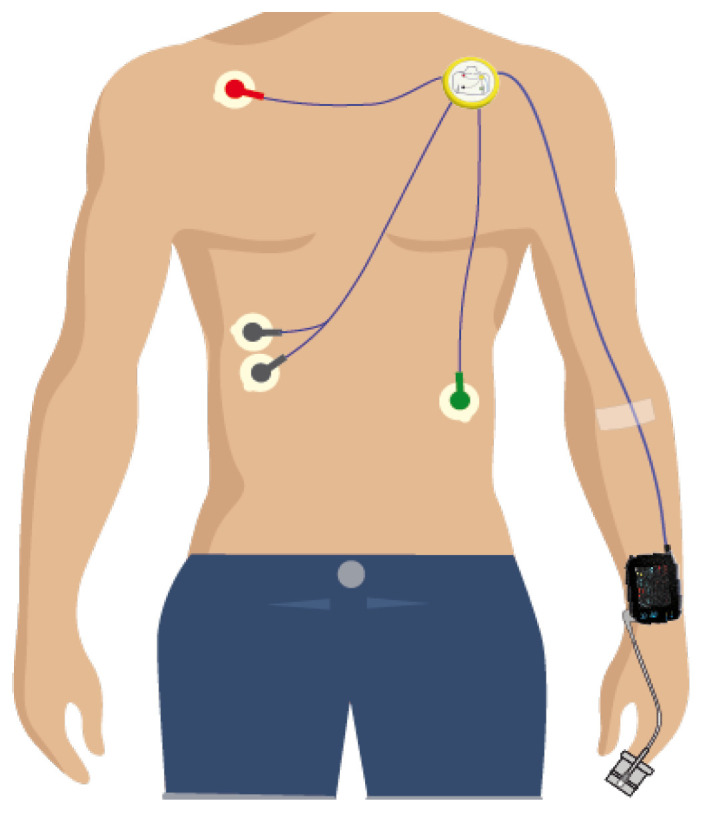
A scheme illustrating the allocation spots of SOMNOtouch-NIBP device and electrodes (Received from Somnomedics http://www.somnomedics.eu).

The used measuring device in this study is SOMNOtouch-NIBP (Figure 1) http://www.somnomedics.eu of the CE and ISO certified company of SOMNOmedics GmbH. The SOMNOtouch-NIBP device is capable of measuring several physiological parameters needed for the estimation of the EWS score in a continuous, noninvasive manner.

### 2.3. Measuring Device

The SOMNOtouch-NIBP device consists of the following sensors:

1. Accelerometer (*x*, *y* and *z*-axis) (m/s${}^{2}$) capable of detecting motion and the position of the body
2. Pulse oximeter to measure the oxygen saturation (%)
3. Photoplethysmograph (PPG) which is used in combination with the ECG to derive cuff-less, noninvasive blood pressure using pulse transit time (PTT) technique.
4. 3-channel ECG from which the heart rate and respiration rate can be derived.
5. Intercostal electromyography (EMG) electrodes to estimate the respiration based on muscle movement.

## 3. Methods

### 3.1. High-Rate EWS Computation

Based on the continuous measurement of the vital signs (heart rate, blood pressure, oxygen saturation Sp$O_{2}$, and respiration rate) the EWS component of each vital sign is computed every minute using a wearable device. However, the used device does not measure body temperature, hence, the EWS component of temperature is excluded from this study. In order to avoid the instantaneous calculation of EWS components of each vital sign, the statistics of the vital signs are calculated within each minute (i.e., mean, median, minimum, and maximum) and based on them the EWS component of each vital sign is computed. The EWS component of each vital sign is computed based on the standard ranges of the EWS used by the hospital of ZOL as listed in Table 1.

Due to motion artefacts and estimation noise (i.e., heart rate (HR) estimation from ECG), the recorded signals have to be denoised. The denoising filter that is used for this approach is a fourth order Butterworth low pass zero-phase filter. The signal is denoised for every minute (60 samples) and the score of the clean signal statistics is computed.

### 3.2. Vital Signs Time-Series Prediction

In this approach, we aim at providing early detection of vital signs’ behaviour based on time series prediction. In contrast with conventional EWS monitoring, the early detection in our approach is based on predicting the future measures of each vital sign for specific prediction horizons, based on the historical measurements of these vital signs. Moreover, we aim to develop a predictive approach that is suitable for online prediction and model personalisation. The online model is required to adapt the prediction given new recorded measurements, and the personalisation is required to consider the individuality of each subject. In order to develop a model with these characteristics, we suggest using a localised learning approach that can handle continuously increasing recorded measurements in addition to online predictions. The chosen localised learning approach is k-nearest neighbours least-squares support vector machines (kNN-LS-SVM) [14,15] that has shown an acceptable performance in different studies tackling the problems of streaming analytics, online prediction, and model personalisation [14,25].

### 3.3. Local Learning of SVMs

In this section, we start by reviewing the main concepts behind SVMs and localised learning approaches for SVMs. Many localised learning algorithms are developed; these algorithms can be divided into two categories. The first category is multiple prototype method which mainly relies on partitioning the input space prior to training the models by either clustering (e.g., k-means) or *Voronoi* partitioning. Therefore, these algorithms provide offline models which do not consider the new streams of data points [15,26,27,28]. A well-known algorithm of that category is Profile SVM (PSVM) [26]. For the second category, some localised learning algorithms rely on weighing functions that train models online. These algorithms are called instance-based learning algorithms (IBL) as locality refers to the training instances in the vicinity of the new test instance. The weighting function can be a square kernel that provides uniform weight to specific neighbouring instances and excluding all other instances such as k-nearest neighbours. Moreover, the weighting function can be a smooth kernel which gives decaying weights to all instances with the distance from the test instance. The disadvantage of the smooth kernel function (e.g., Gaussian and Cosine similarity measure) [18,29,30] is that all training instances or an indefinite number of them are included in the training process but with different weights. On the other hand, the square kernel function (i.e., kNN) provides a controlled computational complexity regardless of the incremental data size in contrast with the other weighing functions. However, the disadvantage of it is the crucial influence of the k number. Therefore, kNN-LS-SVM [14,15,31] is chosen for its advantages of handling streaming data and providing online modelling given the fixed computational complexity. Hence, We will proceed by introducing the hybrid KNN-LS-SVM algorithm for regression.

#### 3.3.1. Support Vector Machines

SVMs are originally presented as binary classifiers, that assign each data instance $x\in R^{d}$ to one of two classes described by a class label y∈{−1,1} based on the decision boundary that maximises the margin $2/{\left||w|\right|}_{2}$ between the two classes. Generally, a feature map $\phi :R^{d}\mapsto R^{p}$ is used to transform the geometric boundary between the two classes to a linear boundary $L:w^{T}\phi \left(x\right)+b=0$ in feature space, for some weight vector $w\in R^{p\times 1}$ and b∈R. The class of each instance can then be found by $y=\mathrm{sgn}\left(w^{⊤}\phi \left(x\right)+b\right)$, where sgn refers to the sign function.

Similar to the classification problems, regression models are obtained via estimating the boundary *L* based on a set of training examples $x_{i}$ (1≤i≤N) with corresponding output values $y_{i}\in R$. In particular, one is interested in parameters w and *b* that minimise a *loss-function*:

(1) $$\min_{w,\;b;\;\xi }\frac{1}{2}w^{⊤}w\;+C\sum_{i=1}^{N}\left(\xi_{i}+{\xi_{i}}^{*}\right),$$

and are subject to:

$$\begin{array}{c} y_{i}-w^{⊤}\phi \left(x_{i}\right)-b\leq \epsilon +\xi_{i},\;i=1,2,\ldots ,N\;,\\ w^{⊤}\phi \left(x_{i}\right)-y_{i}+b\leq \epsilon +{\xi_{i}}^{*},\;i=1,2,\ldots ,N\;,\\ \xi_{i},{\xi_{i}}^{*}\geq 0,\;\;\;i=1,2,\ldots ,N. \end{array}$$

The constant *C* in (1) denotes the *penalty term* that is used to penalise estimation error through the slack variables $\xi_{i}$ and ${\xi_{i}}^{*}$ outside Vapnik ϵ-sensitivity loss function in the opimisation process.

The so-called *kernel-trick* avoids the explicit introduction of a feature map ϕ and implicitly allows for the use feature spaces of infinite dimensionality. A commonly used kernel is given by the Gaussian kernel:

$$k\left(x_{i},x_{j}\right)=\exp\left(\frac{\left|\right|x_{i}-x_{j}{\left|\right|}^{2}}{2\sigma_{0}^{2}}\right),$$

where $\sigma_{0}$ denotes the *kernel bandwidth*. Both $\sigma_{0}$ and *C* can be optimised as hyperparameters in a cross-validation experiment.

LS-SVM’s are obtained by using a least-squares error loss function [32]:

(2) $$\min_{w,\;b;\;e}\frac{1}{2}w^{⊤}w\;+\frac{1}{2}\gamma \sum_{i=1}^{N}{e_{i}}^{2},$$

such that

$$\begin{array}{c} y_{i}=w^{⊤}\phi \left(x_{i}\right)+b+e_{i},\;i=1,2,\ldots ,N. \end{array}$$

where γ is the regularisation constant for LS-SVM. The optimisation procedure introduces errors $e_{i}$ such that $1-e_{i}$ is proportional to the signed distance of $x_{i}$ from the decision boundary. In fact, the non-negative slack variable constraint is removed and the solution of the optimisation problem can be obtained by a set of linear equations, reducing computational effort [32].

#### 3.3.2. KNN-LS-SVM Regressor

Local learning approaches build models that fit the data in the local neighbourhood around a test example and by locally adjusting the model parameters to the properties of the data [18].

While global SVMs consider the same weight for all training instances in the optimisation process (2), local learning approaches allow that the training samples near a test point are more influential than others. Localised learning approaches of SVMs [14] are based on weighting functions $\lambda (x_{s},x_{i})$ that express the similarity between the feature vectors of the *i*-th data point $x_{i}$ and a test instance $x_{s}$. For an LS-SVM, this leads to the following cost function:

(3) $$\min_{w,\;b;\;e}\frac{1}{2}w^{⊤}w+\frac{1}{2}\gamma \sum_{i=1}^{N}\lambda \left(x_{s},x_{i}\right)e_{i}^{2},$$

such that

$$\begin{array}{c} y_{i}=w^{⊤}\phi \left(x_{i}\right)+b+e_{i},\;i=1,2,\ldots ,N. \end{array}$$

Weighted least-squares support vector machines [33] use a similar approach, but here a different weighting function can be used for any given test point $x_{s}$. In this work we will study a binary valued similarity criterion:

$$\lambda \left(x_{s},x_{i}\right)=\left\{\begin{array}{cc} 1 & \mathrm{if}\;||x_{s}-x_{i}{\left|\right|}_{2}\leq r_{s}\\ 0 & \mathrm{otherwise}, \end{array}\right.$$

where $r_{s}$ is the *K*-th smallest distance among $\left\{||x_{s}-x_{i}||;1\leq i\leq N\right\}$. This formulation leads to the hybrid KNN-LS-SVM method that we will apply on the time-series prediction approach. In particular, a regression model is built for each test example using only the training examples located in the vicinity of the test example [15].

KNN-LS-SVM has the additional advantage of sparseness. Indeed, for an LS-SVM or the localised version that uses a continuous similarity function, all input data is required to construct the separating hyperplane [33]. This can be seen by solving the optimisation problem (3). Using the method of the Lagrangian multipliers, we find:

$$\begin{array}{c} L\left(w,b,e;\alpha \right)=\frac{1}{2}{∥w∥}_{2}^{2}+\frac{1}{2}\gamma \sum_{i=1}^{N}\lambda \left(x_{s},x_{i}\right)e_{i}^{2}-\\ \sum_{i=1}^{N}\alpha_{i}\left(w^{⊤}\phi \left(x_{i}\right)+b+e_{i}-y_{i}\right), \end{array}$$

where $\alpha_{i}$ are the *Lagrangian* multipliers. Thus, for a KNN-LS-SVM, the sparseness characteristic is returned to the LS-SVM. In an online learning mode, this sparseness will result in a computational advantage compared to LS-SVM.

**Figure 2:**
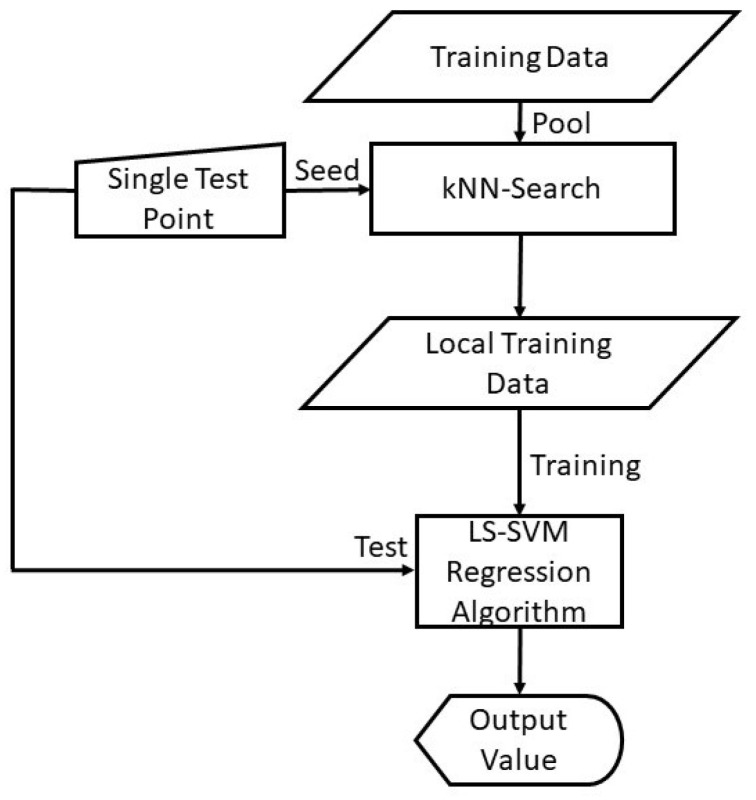
A flow chart illustrating the localised learning algorithm of KNN-LS-SVM for Regression.

As shown in Figure 2, the algorithm of KNN-LS-SVM is implemented as follows:

1. Given a test example $x_{s}$, compute distances to all training examples and pick the nearest *K* neighbours;
2. Train the LS-SVM model with the *K* nearest neighbours.
3. Use the resulting regressor to estimate the output of $x_{s}$.

The parameter *K* and the distance metric (e.g., Euclidean, Mahalanobis, or Chebyshev) are additional hyperparameters next to the kernel width $\sigma_{0}$ and the penalty term γ that are optimised in a cross-validation approach [14]. One challenge faced in finding the nearest neighbour in continuously increasing data pool is the search complexity. However, several advanced search algorithms are developed to reduce this complexity [34].

#### 3.3.3. Prediction-Approach Design

**Figure 3:**
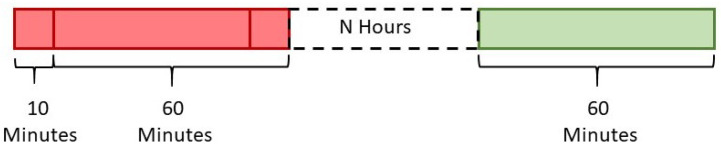
The time-windows for feature extraction (red) comprised of two overlapped windows of 60 min each with an overlap of 50 min resulting in 70 min to predict the statistics of the target window (green) after N hours (e.g., 0,1, or 2 h).

In order to develop a predictive model, several considerations are taken into account that can predict statistics of the vital signs for a specific prediction horizon. Firstly, the time-series prediction problem is formulated as a regression problem whose input comprised of the extracted features from time windows of the measurements of the vital signs (1-h windows). After signal preprocessing, the unified sampling rate for all signals is set to 1 HZ. Hence, it would be more useful to predict the statistics of a time window of a specific size instead of exact samples. After discussions with medical experts, we concluded that predicting the statistics of the vital signs measurements for a prediction horizon of 1 h is of high clinical relevance for the different profiles of patients that we are targeting in this study (i.e., cardiology, dialysis, and postsurgical). Hence, the output of the regression problem is represented by the statistical values (i.e., minimum, maximum, and mean) of the time windows representing the upcoming three consecutive hours (+1, +2, and +3 h) from the end of the feature-extraction period to test the prediction power of our model. For dialysis patients, the prediction will be restricted to the upcoming hour (+1 h) as they are only hospitalised during the dialysis sessions (3–4 h for each session). Furthermore, the next step to define the regression problem is to set the window size, the number of windows, and the overlap percentage between windows from which the features are extracted to be used as input. After testing different window sizes and different overlapping percentages, we found that two overlapping windows of window-size one hour (3600 samples) with an overlap of 50 min (3000 observations) can provide the best possible prediction for the upcoming hour. For practical reasons, this approach is designed to provide a prediction every 10 min based on the previous 70 min recordings as shown in Figure 3. Ultimately, for the train/test division, the leave-one-instance-out approach is used as the training set and includes the data instances of all patients except for one data instance from one patient. Hence, by using kNN-LS-SVM, the nearest points can be from the same subject or similar subjects.

As we have three profiles of patients, namely cardiology, postsurgical, and dialysis patients, it is found to be efficient to test the predictive models on the different profiles individually. However, the main characteristics of the predictive models for each profile will be the same except for the profile of dialysis as the number of observations is relatively low compared to the other wards. Moreover, the extracted features for all profiles are the same, namely minimum, mean, median, maximum, standard deviation, variance, and energy from the denoised signal and its first derivative forming 11 features in total excluding energy of the first derivative. The main difference that is imposed on the predictive model characteristics for dialysis patients is the number of nearest neighbours to train the models locally with. For both cardiology and postsurgical patients, the number of nearest neighbours is 25 data instances; on the other hand, the number of nearest neighbours is 15 for dialysis patients.

Features are extracted from the vital-signs of heart and respiration rate, systolic, diastolic, mean arterial blood pressure, oxygen saturation, and pulse pressure (7 variables). These features are extracted from the time-windows of 1-h (3600 observations), resulting in 77 (11 × 7) dimensions. As shown in Figure 3, the input of the prediction model of kNN-LS-SVM is of two windows, 1-h each, resulting in 144 (2 × 77) input dimensions.

## 4. Results

In this section, the results of implementing and testing the proposed approaches of high-rate EWS computation and vital signs time-series prediction are presented. For the high-rate EWS computation approach, the outcome of each implementation stage is illustrated. On the other hand, the vital-signs prediction approach is tested on the different profiles of patients using the leave-one-instance-out test procedure. Both approaches are applied to 52 cardiology patients at ZOL and 21 at CHU (on average 23 h × 2 days), 7 postsurgical patients at aZM (on avaerage 23 h × 1 day), and 5 dialysis patients at CHU (on average 4 h × 2 sessions). For postsurgical and Dialysis patients, 3 and 2 patients are excluded respectively due to low quality measurements.

### 4.1. High-Rate EWS Computation

**Figure 4:**
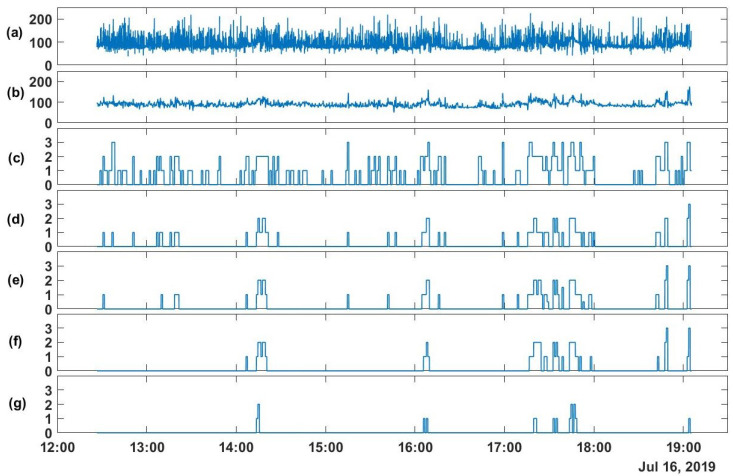
From the heart rate (HR) of a postsurgical patient: (**a**) The raw signal (BPM), (**b**) the denoised signal (BPM), (**c**) the maximum, (**d**) the mean, (**e**) the median, (**f**) the median based on moving median filter, and (**g**) the minimum values of the EWS component of HR for each one-minute segment.

For the first approach, the different statistical values of the monitored vital signs (i.e., maximum, minimum, mean, and median) are computed and then the vital sign score is calculated based on these statistical values. Hence, based on the method elaborated in Section 3.1, the original signal is segmented into nonoverlapping 1-min segments. Each segment is denoised and then the statistical values are computed for the denoised signals (i.e., maximum, minimum, mean, and median). From these statistical values, the vital signs scores are estimated based on the depicted ranges in Table 1. As shown in Figure 4, the results of the different stages of the aforementioned method applied to the heart rate (HR) of a postsurgical patient for approximately 6.5 h of monitoring are depicted.

As mentioned earlier, the recordings of HR in Figure 4 are from a postsurgical patient for the afternoon period. Based on specialists, patients during this period rest after a physiotherapy session in the morning except for the periods of going to the toilet or eating on the table. Therefore, we expect that for those patients, the noise can be due to poor conductivity or local muscular motions.

### 4.2. Vital Signs Time-Series Prediction

For vital signs prediction, the results will be illustrated for the three profiles of patients of cardiology, postsurgical, and dialysis ward. The targeted vital signs are heart rate (HR), systolic blood pressure (SBP), oxygen saturation (Sp$O_{2}$), respiration rate (RR), and pulse pressure (PP) that is derived from the systolic (SBP) and diastolic blood pressure (DBP). The predicted values are the statistical values (minimum, maximum, and mean) of these vital signs for specified future time windows. The upcoming results are based on the leave-one-instance-out test approach.

#### 4.2.1. Cardiology and Postsurgical Patients

**Figure 5:**
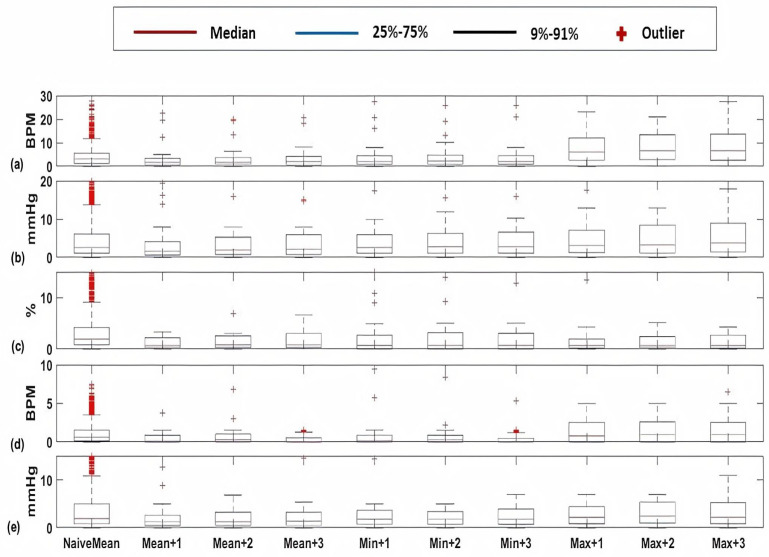
Box-plots of the absolute errors of the Naive predictor at the upcoming hour of the mean value and the proposed algorithm of kNN-LS-SVM of the mean, minimum, and maximum values at the upcoming three hours (+1, +2, +3 h) for the vital signs (**a**) HR (BPM), (**b**) SBP (mmHg), (**c**) Sp$O_{2}$ (%), (**d**) RR (BPM), and (**e**) PP (mmHg) for cardiology patients.

**Figure 6:**
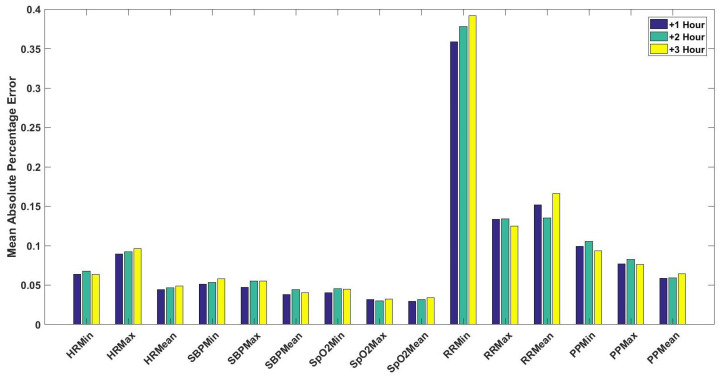
The mean absolute percentage error (MAPE) of the predicted statistical values (i.e., minimum, maximum, and mean) for the vital signs of HR, SBP, Sp$O_{2}$, RR, and PP for the upcoming one, two, and three hours (+1, +2, +3 h) for cardiology patients.

For cardiology and postsurgical patients, the same regression models of kNN-LS-SVM are applied with the same number of k-nearest points (25 data instances). The choice of the number k is optimised based on a cross-validation procedure based on the error performance as explained in [14]. To evaluate the influence of the proposed algorithm, a naive predictor is proposed to be compared with. The naive predictor is assigning the previously observed mean value to the predicted one. The prediction performance of a naive predictor (NaiveMean) in addition to the prediction results for 1-h, 2-h, and 3-h ahead are evaluated using the absolute error as shown in Figure 5. These results are for the vital signs HR (Figure 5a), SBP (Figure 5b), Sp$O_{2}$ (Figure 5c), RR (Figure 5d), and PP (Figure 5e). Furthermore, the mean absolute percentage error (MAPE) of the prediction results are shown in Figure 6 for cardiology patients. Regarding SpO2, its values are normally skewed to be within a range of 20% between 80 and 100%. Therefore, the worst expected value of MAPE is 20%.

**Figure 7:**
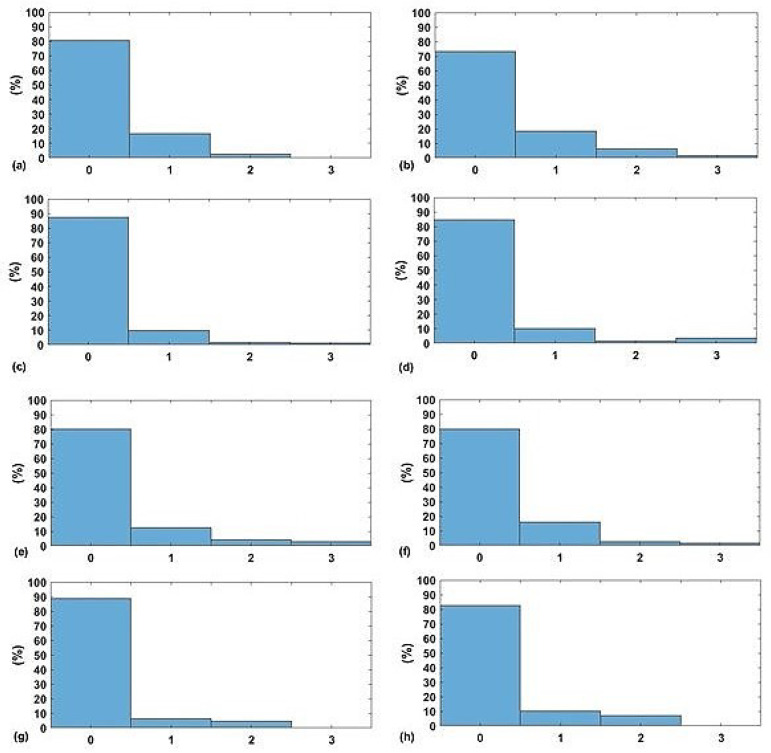
The normalised histogram of EWS components absolute error for both kNN-LS-SVM and Nieve predictors respectively of (**a**,**b**) HR, (**c**,**d**) SBP, (**e**,**f**) Sp$O_{2}$, and (**g**,**h**) RR for cardiology patients.

The next stage is to calculate the EWS for both predicted and actual measures of the vital signs (i.e., HR, SBP, Sp$O_{2}$, and RR). In Figure 7a,c,e,g, the normalised histograms of absolute error between the predicted components of EWS and the actual components of EWS for the four vital signs are depicted. Similarly, normalised histograms of EWS error for naive predictor are shown in Figure 7b,d,f,h.

**Figure 8:**
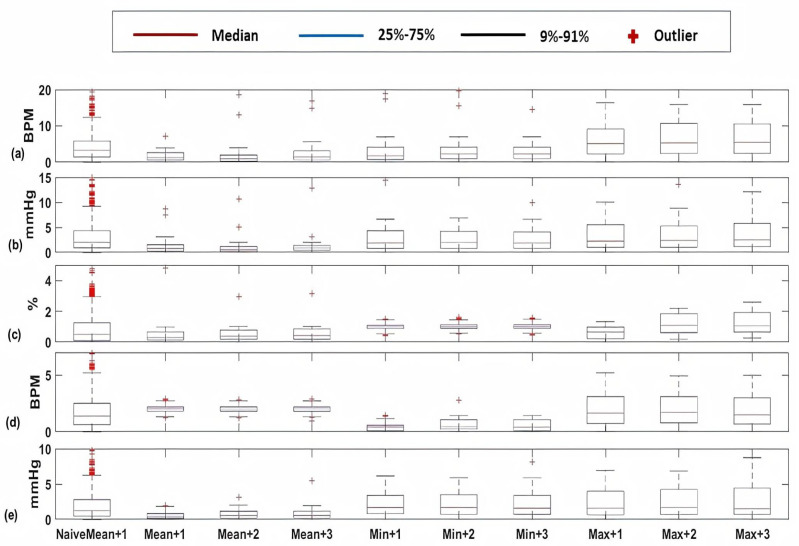
Box-plots of the absolute errors of the Naive predictor at the upcoming hour of the mean value and the proposed algorithm of kNN-LS-SVM of the mean, minimum, and maximum values at the upcoming three hours (+1, +2, +3 h) for the vital signs (**a**) HR (BPM), (**b**) SBP (mmHg), (**c**) Sp$O_{2}$ (%), (**d**) RR (BPM), and (**e**) PP (mmHg) for postsurgical patients.

**Figure 9:**
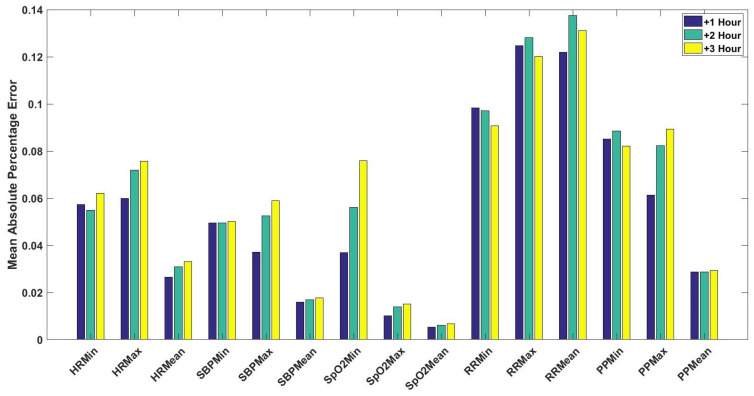
The mean absolute percentage error (MAPE) of the predicted statistical values (i.e., minimum, maximum, and mean) for the vital signs of HR, SBP, Sp$O_{2}$, RR, and PP for the upcoming three hours (+1, +2, +3 h) for postsurgical patients.

**Figure 10:**
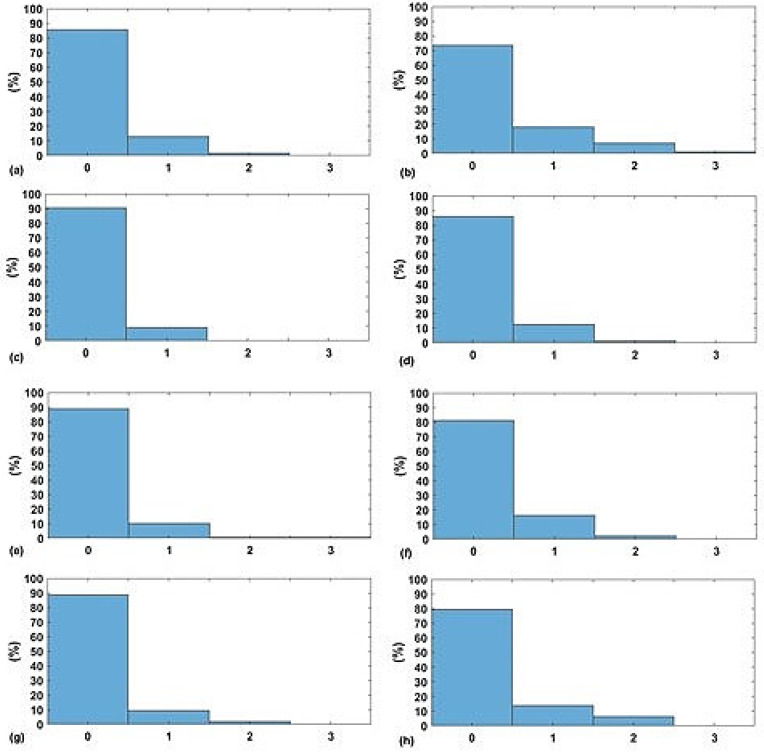
The normalised histogram of EWS components absolute error for both kNN-LS-SVM and Nieve predictors respectively of (**a**,**b**) HR, (**c**,**d**) SBP, (**e**,**f**) Sp$O_{2}$, and (**g**,**h**) RR for postsurgical patients.

Similar to cardiology patients, both absolute error and MAPE results of the vital signs HR (Figure 8a), SBP (Figure 8b), Sp$O_{2}$ (Figure 8c), RR (Figure 8d), and PP (Figure 8e) for postsurgical patients are depicted in Figure 8 and Figure 9. Moreover, the normalised histograms of the EWS components absolute error of HR, SBP, Sp$O_{2}$, and RR respectively are shown in Figure 10a,c,e,g. Moreover, normalised histograms of EWS error for naive predictor are shown in Figure 10b,d,f,h).

#### 4.2.2. Dialysis Patients

**Figure 11:**
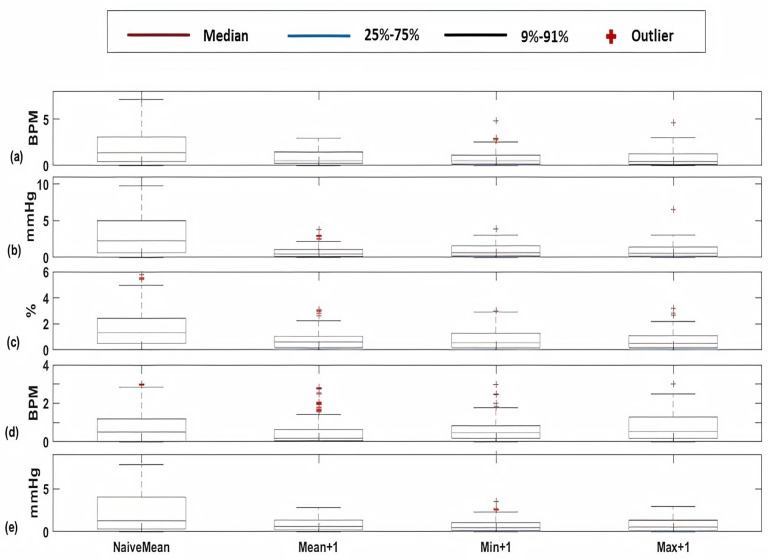
Box-plots of the absolute errors of the Naive predictor of the mean value and the proposed algorithm of kNN-LS-SVM of the mean, minimum, and maximum values at the upcoming hour for the vital signs (**a**) HR (BPM), (**b**) SBP (mmHg), (**c**) Sp$O_{2}$ (%), (**d**) RR (BPM), and (**e**) PP (mmHg) for Dialysis patients.

**Figure 12:**
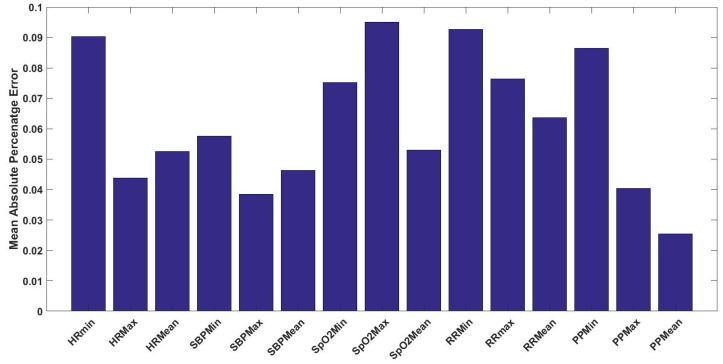
The mean absolute percentage error (MAPE) of the predicted statistical values (i.e., minimum, maximum, and mean) for the vital signs of HR, SBP, Sp$O_{2}$, RR, and PP for the upcoming hour for dialysis patients.

**Figure 13:**
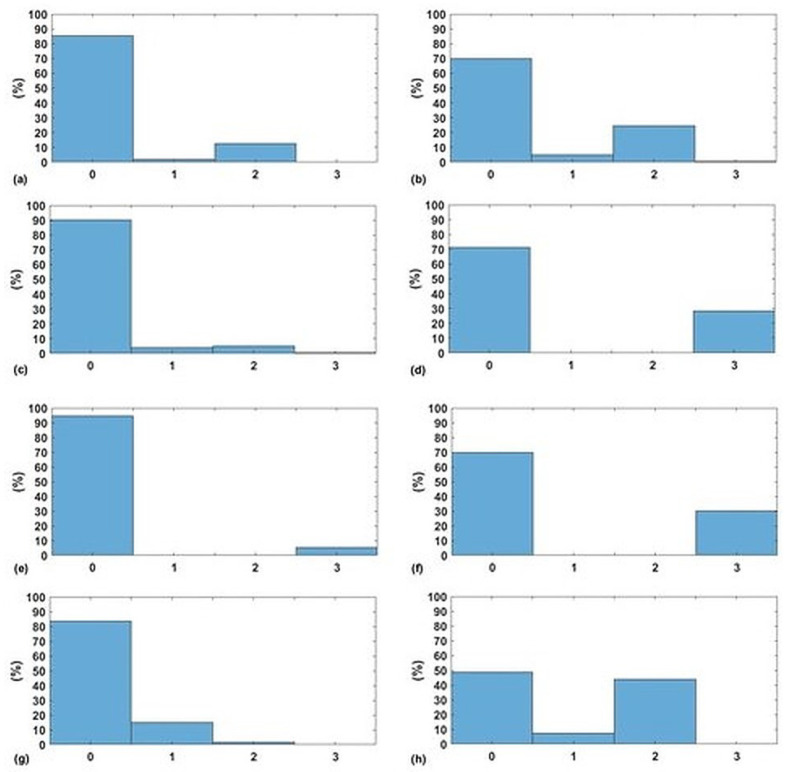
The normalised histogram of EWS components absolute error for both kNN-LS-SVM and Nieve predictors respectively of (**a**,**b**) HR, (**c**,**d**) SBP, (**e**,**f**) Sp$O_{2}$, and (**g**,**h**) RR for Dialysis patients.

For dialysis patients, the predictive models are slightly different from the previous models due to the disease characteristics of these patients. As dialysis patients at haemodialysis are regularly in-hospital (i.e., at least 3 days per week), with a duration of four hours per session, the prediction is only applied for the next hour instead of three hours. Furthermore, it is found that the optimal k-number of the nearest neighbours is 15 data instances based on the cross-validation procedure. The prediction error performance, absolute error, and MAPE, for the statistical values of the vital signs for the upcoming hour are depicted in Figure 11 and Figure 12 respectively. Ultimately, the normalised histograms for the vital signs components of EWS absolute error are shown in Figure 13a,c,e,g). Finally, normalised histograms of EWS error for naive predictor are shown in Figure 13b,d,f,h).

After showing the results of the three profiles of patients it is noticed that for both cardiology and postsurgical patients, the significance was achieved at α of 0.01 resulting in *p*-values approximately zeros for all vital signs. The significance is achieved for dialysis patients at α of 0.05 with *p*-values 0.05, 0.06, 0.064, 0.042, and 0.033 for HR, SBP, Sp*O*_2_, RR, and PP respectively.

## 5. Discussion

Several studies investigated vital sign data collected from continuously monitored hospitalised patients, more in particular, intensive care unit (ICU) patients. For this purpose, publicly available datasets such as MIMIC have been used and of which different versions are available (MIMIC, MIMIC II, and MIMIC III) [35,36]. However, continuously monitoring vital signs requires expensive cumbersome devices at the ICU [1,37]. Many of these studies target the early detection of vital signs’ deterioration based on novelty detection approaches [38,39,40,41,42]. In our study, monitoring is performed with medically approved wearable technology. Such devices have the advantage of being relatively cheaper and they allow a mobile and portable monitoring approach. Moreover, this study assesses the vital signs by time-series prediction and real-time estimation of EWS components from each vital sign. It is worth mentioning that the used EWS standard thresholds are from the hospital of ZOL; however, our algorithms can be adapted to any EWS standards of any hospital.

As shown in Section 4, the first approach provides a high- rate real-time estimation of the EWS components obtained from the vital signs’ data. (HR, SBP, Sp$O_{2}$, and RR). Real-time EWS components are obtained by estimating vital signs’ scores every 60 s after signal preprocessing. Such real-time vital signs assessments are already possible for critically ill patients at ICUs that are monitored by expensive equipment and are restrained to their bed. For general-ward patients, however, such real-time EWS components estimation is not performed in clinical practice due to the restrictions of, among others, manual and infrequent measurements by nurses. Here, we demonstrate that frequent measurements using wearables for general-ward patients allow real-time estimation of EWS as well. To illustrate the implementation of the first approach, a representative example of a postsurgical patient is used. As shown in Figure 4, the raw signal (Figure 4a) of HR is noisy due to motion artefacts and possible conductivity issues. Hence, it is important to denoise the raw signal on segments of one-minute each to discover the underlying trend and deviations from it. For that purpose, a Butterworth low pass filter zero-phase (fourth order) is used to denoise the signals with cut-off frequencies between 0.03 and 0.04 Hz for the different signals. The signal shown in (Figure 4b) is a sequence of denoised nonoverlapping segments (one-minute each). After obtaining a clean signal, the EWS can be easily calculated given the hospital standard thresholds for each vital sign as shown in Table 1. For practical reasons, we find that instead of providing sample-by-sample EWS, we can provide the EWS components of the statistical values of the vital sign within each segment. Therefore, within each segment we provide the maximum, mean, median, and minimum observed vital sign score as shown in Figure 4. We notice from these observed vital signs scores that the variance within the same segment is reasonable as the difference between the minimum and maximum scores for the same segment is mostly unity, taking into account that the EWS ranges are continuous without guard-intervals. Moreover, both the mean and the median are very similar which indicates the absence of outliers and consistency within each segment. Furthermore, in Figure 4f, we show the median EWS component of HR based on moving median filter. This filter is implemented by extracting the median value within a moving window of one minute width and shifting with one sample. Compared to Figure 4e, the moving median filter is missing several details, especially those that lead to unity EWS component of HR.

For the time-series prediction approach, the results in Section 5 show that the prediction of vital signs based on historical values of these vital signs is feasible considering the balance between the feature-extraction horizon (70 min) and the prediction horizon (1–3 h). As mentioned earlier, there are three profiles of patients that are monitored and analysed.

Firstly, Figure 5 shows significant difference between a naive predictor—that just predicts the next hour value similar to the most previous measure—and the kNN-LS-SVM predictor. This significance is approved by the paired t-test for all vital signs for different profiles. For the two profiles cardiology and postsurgical patients, the parameters of the kNN-LS-SVM are set to the same values. In particular, the number of nearest neighbours is set to k=25. In Figure 5 and Figure 6, the absolute error and MAPE for cardiology patients are illustrated respectively. It is observed that the extreme values (minimum and maximum), especially maximum, have higher errors than the mean. This is due to the high variability of the extreme values from one window to another especially when the window size is one hour. Moreover, the parameters with high average amplitude (e.g., 124 mmHg for SBP) have higher absolute error than that of RR which has low average amplitude (approximately 14 BPM), in contrast with results of MAPE since the opposite is observed. Furthermore, it is observed that the maximum value of HR has the largest absolute error (mean, median, and standard deviation) which may indicate the high fluctuation of the extreme values of HR. On the other hand, the highest MAPE is observed by the minimum value of RR as the minimum values can be approximately 5 BPM, hence having an absolute error of 2 leads to 40% MAPE. Another important observation is that the error performance evolution from one prediction horizon to another is at maximum 1.8,2.5% for absolute error (mean) and MAPE respectively. In Figure 7, the normalised histograms of EWS components absolute error of the vital signs HR, SBP, Sp$O_{2}$, and RR are depicted. These histograms show the dominance of zero error with minimum 80% (HR and Sp$O_{2}$) and maximum 88% (RR) but the unity error of maximum 14% (HR) and minimum 7% (RR). The rest of the possible absolute error values (2 and 3) are minority with maximum 5% (Sp$O_{2}$).

For postsurgical patients, as shown in Figure 8 and Figure 9, we notice that the fluctuation of absolute error of blood pressure parameters (SBP and PP) in addition to HR is low compared to cardiology patients. Moreover, the MAPE for all vital signs is less than that of cardiology patients. In Figure 10, the normalised histograms of EWS components absolute error show a higher dominance of zero error with minimum 87% (HR) and maximum 90% (SBP). However, the unity error of maximum 14% (HR) and minimum 10% (RR). The rest of the possible absolute error values (2) are minority with maximum 2% (Sp$O_{2}$) and now error of 3 is observed.

For dialysis patients, some features about the model and the approach are modified to meet the characteristics of this profile of patients. As mentioned earlier, this profile of patients is normally hospitalised and monitored during the dialysis sessions (3–4 h/session for haemodialysis). Hence, it is found that one-hour prediction is sufficient for that profile also the optimal number of nearest points is 15 data-points for the kNN-LS-SVM regression model. As shown in Figure 11 and Figure 12, the overall error performance for +1 h prediction is comparable to that of the other profiles of patients either for absolute error or MAPE. One remark is that, in contrast with the other profiles, the prediction error for the minimum value for HR and SBP is higher than that of the maximum. That may indicate that extremely low values of these vital signs for those patients are less predictable than the other values. One general observation for the different profiles of patients is that the error performance is not systematically degrading with increasing the prediction horizon. A possible interpretation is regarding the temporal resolution provided by the window size. Hence, increasing the window-size can eliminate this observation, but this will lead to decreasing our data size, which affects the performance. Therefore, we keep the window-size of one hour, which provides the best error performance together with keeping a proper data size. In Figure 13, the normalised histograms of EWS components absolute error show a maximum zero error of 94% (Sp$O_{2}$) and minimum 83% (RR). The unity error is observed, in contrast with the other profiles, less than that of 2 (HR, SBP, and Sp$O_{2}$) and 3 (Sp$O_{2}$). This can be due to the relatively smaller size of data compared to the other profiles, hence, the distribution became not skewed normal as expected. Moreover, the errors of 2 and 3 are mainly observed in the vital signs of Sp$O_{2}$ and RR. This can be interpreted as a result of the narrow EWS ranges for both vital signs as shown in Table 1.

From the obtained histograms, we have noticed that the observed EWS error has in few cases the value of 3 especially for the vital signs of SBP and Sp$O_{2}$. By investigating the instances that have an EWS error of 3 for SBP, we found that this error of 3 is due to noise. This conclusion is drawn from observing abrupt changes in the EWS from 0 to 3 or vise versa. Moreover, there are only 10 min between two consecutive observations representing windows of one hour each, with 50 min overlap. Therefore, we conclude that these extreme EWS errors are more due to artefacts and their associated noise than physiological behaviour. On the other hand, the EWS error of 3 for SpO2 is either due to the small range of Sp$O_{2}$ between scores 0 and 3 (90–96%) as shown in Table 1 or due to noise.

A general observation regarding histograms (Figure 7, Figure 10, and Figure 13) of the Naive predictor EWS error is that the performance for both cardiology and postsurgical patients is worse than our proposed model but not radically worse. This is due to the high percentage (>80%) of the observed 0 EWS for all vital signs along the period of recording. On the other hand, the histogram of the Naive predictor for dialysis patients is much worse than that of our proposed model. This can be because of the more dynamic behaviour of the vital signs during the dialysis sessions. Hence, the variability from one observation to another can mislead the Naive predictor.

Ultimately, it is noticed that the locally selected instances for the three profiles of patients are not all from the same patient. Moreover, more than 50% of the nearest neighbours are from different subjects. This shows the advantage of using our localised learning approach that can provide an accurate online performance using same-subject data and together with the most similar instances of other subjects. Therefore, the concept of model personalisation is extended to include similar subjects in addition to same-subject data.

After showing the results for each of the considered profile of the patients, we discuss our predictive model’s error performance in the light of the study of Shiyu et al. [13] which is the most relevant one according to the authors. However, the differences between the two studies have to be considered regarding the prediction horizon and the nature of the predicted values (i.e., exact or statistical). In their study, Shiyu et al. show that their best results of the boosting generative LSTM approach for vital signs prediction are 7.41% for HR and 6.17% for SBP of MAPE. These prediction results are for 20 min prediction horizon only. On the other hand, our prediction results are not for exact values but for statistical values of time windows. However, for comparison purposes, the mean values of the future time windows are used considering the average prediction errors of the three prediction horizons (i.e., +1, +2, +3 h). For HR, the MAPE’s are 4.5, 3.1, and 6.3% for cardiology, postsurgical, and dialysis patients respectively. For SBP, the MAPE’s on average are 4.4, 1.8, and 4.6% for cardiology, postsurgical, and dialysis patients respectively. Based on these results, we claim that our approach is promising given the prediction horizons and the error performance. Moreover, a more recent study [43] is presenting an LSTM based predictive model to predict the vital signs of hospitalised patients. However, the used time windows are relatively small since the best results obtained with 1-min windows and the number of admissions needed fro the best performance is 2500 admissions. The developed deep network requires 22 h for training and the best error performance obtained is 81%. Ultimately, as mentioned earlier, the vital sign of body temperature, one of the five main EWS components, is missing in this study as the used wearable device does not provide it. However, our resulting outcome of continuous monitoring and time-series prediction of EWS components of the other vital signs is still informative. These components can be provided to the medical staff in an aggregated form to give the complete EWS once the body temperature is available. Moreover, the body temperature of general wards patients is not as dynamic as the other vital signs (e.g., HR). Hence, we do not expect any difficulty to predict it once its values are provided.

## 6. Conclusions

In this study, we proposed two approaches for high-rate EWS computation and time-series prediction based on vital signs measured on hospitalised patients using a wearable device in a cardiology, postsurgical, and dialysis ward. The first approach is the estimation of the high-rate (every minute) scores of the statistical values of the measured vital signs of HR, SBP, RR, and Sp$O_{2}$, for each one-minute segment, based on the depicted thresholds in Table 1. On the other hand, the second approach comprises predictive models by which the future values of monitored vital signs in addition to the pulse pressure (PP) are predicted. This approach is designed to provide a prediction result every ten minutes. The used technique is the hybrid machine learning algorithm of kNN-LS-SVM. The predicted values are the statistical values (i.e., minimum, maximum, and mean) of the future time-windows within specified prediction horizons. The used prediction horizons for both cardiology and postsurgical patients are 1-, 2-, 3-h ahead (+1, +2, +3 h). For dialysis patients, the prediction horizon is only the upcoming hour due to the relatively short stay. The prediction performance is evaluated based on the error metrics of the absolute error and mean absolute percentage error (MAPE), and the followed test procedure is that of leave-one-out. The prediction error performance shows outperforming results compared to a naive predictor as well as to the best performing and most recent state of the art [13]. Hence, we conclude that our prediction approach can provide an acceptable prediction performance that can add a predictive insight to the medical staff monitoring the health status of the monitored patients. Furthermore, the prediction approach can handle both online prediction and streaming analytics as a main feature of the used method of kNN-LS-SVM in addition to model personalisation. For the high-rate estimation of EWS, the proposed approach shows the possibility to provide an online estimation of the EWS based on the real-time signal preprocessing and vital sign score computation. Ultimately, continuous vital signs monitoring using wearable technologies can provide a real-time estimation of the EWS and time-series prediction using a localised learning algorithm. In this way, the combination of wearables and machine learning can contribute to a more accurate monitoring of patients in hospital settings.

For future work, we would suggest investigating the patients taking into consideration their status during their hospitalisation stay. For instance, we would expect clinicians to give a label to the analysed patients regarding their likelihood of deterioration. In this case, we would test our models specifically on those critical cases to evaluate the efficiency of our models with such cases.

## Figures and Tables

**Table 1:** Early Warning Scores system based on Ziekenhuis Oost-Limburg (ZOL) Hospital.

SCORE	3	2	1	0	1	2	3
Temperature (${}^{\circ }$C)		<35.1	35.1–36.5	36.6–37.5	>37.5		
Heart Rate (BPM)		<40	40–50	51–100	101–110	111–130	>130
Respiration Rate (BPM)		<9		9–14	15–20	21–30	>30
Oxygen Saturation (%)	<91	91–93	94–95	>95			
Systolic Blood Pressure (mmHg)	<70	70–80	81–100	101–180	180–200	>200	
